# Supplementary material for: WordCommentsAnalyzer: A windows software tool for qualitative research
Source: F1000Res. 2018 Sep 4;7:536. Originally published 2018 May 3. [Version 2] doi: 10.12688/f1000research.14819.2 (PMC6137411; doi:10.12688/f1000research.14819.2)
Supplement: Supplementary file 3 [file f1000research-7-17614-s0002.tgz › b2a79496-4ada-47c4-9682-a99ce867836d.zip!/Eight Iranian Health Journals/Journal of Health System Research _10.docx]

© Journal of Health System Research . This work is licensed under CC BY-NC 4.0 (https://creativecommons.org/licenses/by-nc/4.0/).

Volume & Issue: دوره 11، شماره 4، 1394

Title: بررسی فراوانی آسیب‌های شغلی و آگاهی از احتیاط‌های استاندارد در کارکنان آزمايشگاه و پرستاران

http://hsr.mui.ac.ir/index.php/jhsr/article/view/1371

مقدمه: بیماری‌های هپاتیت B و C و عفونت HIV (Human immunodeficiency virus) در کشور ما از جمله مشکلات بهداشتی محسوب می‌شود. برای کاهش مواجهه با آسیب‌های شغلی‌ که باعث انتقال این عفونت‌ها می‌شوند، اصولی طراحی شده است که با آگاهی از آن‌ها می‌توان تا حد بالایی از مواجهه با اين آسیب‌ها پیشگیری نمود. پژوهش حاضر با هدف تعیین میزان آسیب‌های شغلی و تعيين آگاهی از احتیاط‌های استاندارد در کارکنان آزمايشگاه و پرستاران شهرستا‌ن‌های دهاقان و شهرضا طراحی شد. روش‌ها: این پژوهش مقطعی بر روی 77 نفر از پرستاران و کارکنان آزمایشگاه شاغل در مراكز دولتي وابسته به دانشگاه علوم پزشكي اصفهان که به روش سرشماری انتخاب شدند، صورت گرفت. جهت سنجش آگاهی از احتیاطات استاندارد و تعیین میزان آسیب‌های شغلی، از پرسش‌نامه محقق ساخته استفاده گردید. داده‌های جمع‌آوری شده با استفاده از شاخص‌های توصیفی و آزمون‌های 2c و Mann–Whitney در نرم‌افزار SPSS مورد تجزیه و تحلیل قرار گرفت. یافته‌ها: 4/84 درصد از نمونه‌ها دارای سطح آگاهی ضعیف و 6/15 درصد دارای سطح آگاهی متوسط بودند. 5/80 درصد از افراد، هر سه نوبت واکسن هپاتیت B را به طور کامل دریافت کرده بودند. ارتباط آماری معنی‌داری بین ایمن‌سازی علیه هپاتیت B و جنسیت مشاهده شد (05/0 > P). بعد از تلقیح واکسن، 61 درصد نمونه‌ها اقدام به سنجش تیتر آنتی‌بادی خود کرده بودند. شایع‌ترین آسیب شغلی، فرورفتن سر سوزن (Needle stick) در دست بود؛ به طوری که 6/50 درصد از آزمودنی‌ها فرورفتن سر سوزن در دست را تجربه کرده بودند. نتیجه‌گیری: با توجه به پایین بودن سطح آگاهی افراد مورد مطالعه و بروز مکرر آسیب‌ها و صدماتی همچون فرورفتن سوزن در دست، بریدن و خراشیدن پوست و... در آن‌ها، لزوم برنامه‌ريزي منسجم و مداوم براي آموزش افراد در رابطه با اصول احتياط‌هاي استاندارد ضروری به نظر می‌رسد.

Title: ارتباط بین علایم اختلالات روانی و فرسودگی شغلی در کارکنان بهداشتی و درمانی

http://hsr.mui.ac.ir/index.php/jhsr/article/view/1875

مقدمه: پژوهش حاضر با هدف، تعیین رابطه بین علایم اختلالات روانی با فرسودگی شغلی در کارمندان دانشگاه علوم پزشکی شیراز انجام شد. روش‌ها: این پژوهش یک مطالعه مقطعی بود. تعداد 300 کارمند با روش نمونه‌گیری تصادفی ساده انتخاب شدند. علایم اختلالات روانی، متغیرهای پیش‌بین و فرسودگی شغلی، متغیرهای ملاک پژوهش حاضر بودند. نشانگان اختلالات روانی تجدید نظر شده و پرسش‌نامه شغلی Maslach (Maslach Burnout Inventory یا MBI)، ابزارهای جمع‌آوری داده‌ها بود. یافته‌ها: رابطه مثبت و معنی‌داری بین فرسودگی شغلی و کلیه نشانه‌های اختلالات روانی وجود داشت. پرخاشگری عامل پیش‌بینی کننده معنی‌دار خستگی هیجانی (52/0 = r، 001/0 > P) و مسخ شخصیت (41/0 = r، 010/0 = P) بود. از بین علایم اختلالات روانی، پرخاشگری (45/0 = r، 030/0 = P) و افکار پارانویید (45/0 = r، 001/0 > P) توانسته بودند به طور مثبت و معنی‌داری، بعد کاهش عملکرد را پیش‌بینی نمایند. نتیجه‌گیری: در میان علایم اختلالات روانی، پرخاشگری مهم‌ترین عامل پیش‌بینی کننده فرسودگی شغلی محسوب می‌شود. به نظر می‌رسد برنامه‌ریزی برای خدمات آموزشی، سازمانی و مداخلات روانی بر اساس نتایج مطالعه حاضر، می‌تواند نقش مهمی در ارتقای سلامت روان و رضایت شغلی کارکنان دانشگاه علوم پزشکی داشته باشد.

Title: بررسی تأثیر دوره‌های زیستی بر وقوع حوادث شغلی در صنایع فلزی

http://hsr.mui.ac.ir/index.php/jhsr/article/view/2236

مقدمه: بيوريتم يكي از جديدترين مباحث در زمينه ارگونومي ذهنی است كه با شناخت جنبه‌هاي جسمي، عاطفی و ذهنی افراد مي‌تواند در بالا بردن ميزان رضايت شغلي مؤثر باشد و از بروز بسياري از حوادث در محيط كار جلوگيري نمايد. مطالعه حاضر با هدف بررسی تأثیر دوره‌های زیستی بر وقوع حوادث شغلی در صنایع فلزی انجام شد. روش‌ها: این مطالعه به صورت مقطعی و توصیفی- تحلیلی بر روی 120 حادثه شغلی که به نوعی افراد در وقوع حادثه تأثیر داشتند، صورت گرفت. در نهایت، با استفاده از اطلاعات به دست آمده از برگه گزارش حوادث و با استفاده از نرم‌افزار Easy biorhythm calculator، ارتباط بین تأثیر دوره‌های زیستی و وقوع حوادث شغلی مورد بررسی قرار گرفت. یافته‌ها: دوره‌های زیستی بر وقوع حوادث شغلی تأثیر‌گذار بود؛ به طوری که روزهای بحرانی و بخش منفی چرخه جسمانی (فیزیکی) تأثیر بیشتری در وقوع حوادث نسبت به چرخه‌های ذهنی و عاطفی داشت. نتیجه‌گیری: نتایج تحقیق نشان داد، در مواقعی که افراد از لحاظ جسمانی (فیزیکی) در بخش منفی و روزهای بحرانی دوره‌های زیستی قرار داشتند، وقوع حادثه بیشتر بود؛ به این دلیل که کار مورد نظر فراتر از توانایی افراد برای انجام دادن آن بود و سبب وقوع حادثه گردیده بود.

Title: بررسی آلودگی صوتی و شاخص صدای ترافیک با استفاده از سیستم اطلاعات جغرافیایی در خیابان‌های اصلی شهر کاشان

http://hsr.mui.ac.ir/index.php/jhsr/article/view/2544

مقدمه: مطالعات نشان دادند که آلودگی صوتی بر سلامت انسان تأثیرات مضری دارد. صدای ناشی از ترافیک شهرها، مهم‌ترین منبع آلودگی صوتی به شمار می‌رود. هدف مطالعه حاضر، تعیین آلودگی صوتی خیابان‌های اصلی شهر کاشان با استفاده از سیستم اطلاعات جغرافیایی (Geographic information system یا GIS) بود. روش‌ها: 180 مورد اندازه‌گیری صدا در 20 منطقه از خیابان‌های اصلی شهر کاشان به وسیله صداسنج مدل 440CEL- و با روش استاندارد 1996 ISO انجام گرفت. شاخص‌های تراز معادل صدا (LAeq)، تراز آماری صدا، تراز حداقل و حداکثر صوت برای هر اندازه‌گیری در 3 نوبت صبح (10:30-7)، ظهر (15:30-12) و شب (21:30-18) تعیین گردید. شاخص صدای ترافیک محاسبه و بر روی نقشه‌های پهنه‌بندی GIS رسم شد. یافته‌ها: میانگین و انحراف معیار LAeq برای کل اندازه‌گیری‌ها برابر با (dB)A 1/1 ± 2/77 و میانگین شاخص آلودگی صوتی ناشی از ترافیک کل اندازه‌گیری‌ها برابر با (dB)A 5/68 بود. تفاوت معنی‌داری بین ایستگاه‌های مورد مطالعه برای شاخص صدای ترافیک مشاهده گردید (001/0 > P)، اما تفاوت معنی‌داری بین این ایستگاه‌ها در مورد LAeq مشاهده نشد (140/0 = P). نتیجه‌گیری: آلودگي صوتي در شهر کاشان بالاتر از استاندارد سازمان حفاظت محيط زيست ايران بود. علت اصلی آلودگی صوتی این شهر، وسایل نقلیه موتوری می‌باشد که اقدامات کنترلی در این زمینه لازم است.

Title: بررسی نقش ایزولاتور صفحه‌ای در کاهش میزان ارتعاشات منتقله در بدنه کانال و پایه فن سیستم تهویه

http://hsr.mui.ac.ir/index.php/jhsr/article/view/2068

مقدمه: کاربرد سیستم‌های تهویه در صنایع و محیط‌های کاری با هدف بهبود شرایط محیطی برای کارکنان انجام می‌شود و عدم توجه به طراحی صحیح اجزای مختلف سیستم تهویه و روش‌های کنترلی مناسب، باعث تولید صدا و ارتعاش در بیشتر اجزای آن می‌گردد. بدین منظور، مطالعه حاضر با هدف تعیین نقش ایزولاتور صفحه‌ای در کنترل ارتعاشات سیستم تهویه برای کاهش ارتعاشات سیستم تهویه و همچنین، کاهش صدای سیستم انجام گردید. روش‌ها: در این مطالعه تحلیلی، سیستم مورد نظر از نوع HVAC (Heating, ventilating and air conditioning) بود که دارای کانالی از جنس گالوانیزه و فن ‌محوری بود. سنجش ارتعاش در بدنه کانال و پایه فن صورت گرفت و کاهش ارتعاشات منتقل شده پس از نصب و به‌ کارگیری ایزولاتور بررسی گردید. یافته‌ها: میزان کاهش سرعت ارتعاشی فن در سرعت 2580 دور بر دقیقه، 61 درصد به دست آمد و بیشترین تأثیر ایزولاتور ارتعاشی نیز در همین دور از فن بود. همچنین، در سرعت‌های بالا ایزولاتور تأثیر بسیار بهتری در کاهش سرعت ارتعاشی داشت و در سرعت‌های 2200 دور بر دقیقه و پایین‌تر، کاهش محسوسی در تأثیر ایزولاتور در کاهش سرعت ارتعاشی مشاهده شد. نتیجه‌گیری: استفاده از ایزولاتور لاستیکی برای ماشین‌آلات کوچک مانند فن‌ها در سیستم تهویه، کارایی خوبی دارد که بر اساس بار وارد شده، جابجایی استاتیکی و میزان فشردگی ماده سازنده، می‌توان از آن‌ها به عنوان وسیله‌ مؤثری در کاهش ارتعاش استفاده شود.

Title: بررسی الگوی مصرف نوشیدنی‌های انرژی‌زا و فاکتورهای مرتبط در دانشجویان

http://hsr.mui.ac.ir/index.php/jhsr/article/view/2258

مقدمه: استفاده نوشیدنی‌های انرژی‌زا با رفتارهای پرخطری از جمله مصرف سیگار و الکل رابطه مستقیم دارد. هدف مطالعه حاضر، بررسی الگوی مصرف نوشیدنی‌های انرژی‌زا و فاکتورهای مرتبط با آن در میان دانشجویان بود. روش‌ها: این پژوهش توصیفی- مقطعی، بر روی دانشجویان دانشگاه علوم پزشکی اصفهان انجام گردید که در سال 92-1391 تحصیل می‌کردند. 373 دانشجو از طریق نمونه‌گیری تصادفی و طبقه‌ای دو مرحله‌ای وارد مطالعه شدند. ابزار جمع‌آوري اطلاعات، پرسش‌نامه محقق ساخته مشتمل بر 2 بخش اطلاعات جمعیت‌شناختی و رفتارهای مصرف شامل نوشیدنی‌های سرد، سیگار و مشروبات الکلی بود. یافته‌ها: مصرف نوشیدنی‌های انرژی‌زا در 7 درصد افراد مورد مطالعه گزارش گردید. ضریب همبستگی Pearson بین مصرف نوشیدنی‌های انرژی‌زا با مصرف آب میوه‌های بسته‌بندی شده، نوشیدنی‌های گازدار، سیگار و مشروبات الکلی ارتباط معنی‌داری را نشان داد (001 > P). همچنین، 3/71 درصد افراد مورد مطالعه، نوشیدنی‌های انرژی‌زا را بدون ذکر علت خاصی مصرف می‌کردند. نتیجه‌گیری: با توجه به ارتباط میان مصرف نوشیدنی‌های انرژی‌زا و شانس بیشتر درگیر شدن به رفتارهای پرخطری مانند مصرف سیگار و مشروبات الکلی، ضرورت اجرای مداخلات آموزشی در این زمینه بر روی دانشجویان احساس می‌شود.

Title: تأثیر غلظت اتیلن‌دی‌کلراید بر روی متان‌سازی بیومس بی‌هوازی

http://hsr.mui.ac.ir/index.php/jhsr/article/view/2232

مقدمه: مطالعه حاضر با هدف بررسی تأثیر بازدارندگی اتیلن‌دی‌کلراید (Ethylene dichloride یا EDC) بر روی تجزیه زیستی از طریق آزمون فعاليت متان‌سازي ويژه (Specific methanogenic activity یا SMA) بيومس بي‌هوازي انجام شد. روش‌ها: تعداد 40 آزمون SMA بیومس بی‌هوازی به روش ناپيوسته و در ويال‌هاي شيشه‌اي 120 میلی‌لیتری صورت گرفت. 17 درصد از حجم هر ویال به بیومس، 66 درصد به سوبسترا و 17 درصد به تجمع بیوگاز اختصاص یافت. مدت هر batch، 10 روز به طول انجاميد. متان توليد شده به وسيله جايگزيني گاز با محلول 2 نرمال هیدروکسید پتاسیم (KOH) به عنوان جاذب 2 CO اندازه‌گيري شد. آزمایش اکسیژن مورد نیاز شیمیایی (COD یا Chemical oxygen demand) قبل و بعد از هر تست بر روی نمونه‌ها انجام شد. تغییرات دمای آزمایش C° 2 ± 35 و pH در محدوده 7 حفظ شد. COD ورودی نیز برای هر ویال در ابتدا و انتها سنجیده می‌شد. اتیلن‌دی‌کلراید با استفاده از دستگاه کروماتوگرافی گازی مجهز به آشکار‌ساز اسپکترومتری جرمی (GC-MS) در ابتدا و انتهای دروه آزمایش آنالیز گردیدد. یافته‌ها: در غلظت‌های 500، 1000، 5000 و20000 میلی‌گرم در لیتر اتیلن‌دی‌کلراید، کاهش تولید متان به ترتیب به میزان 6/12، 9/9، 98/2 و 86/0 ميلی‌ليتر متان به ازای هر گرم VSS (Volatile suspended solids) در روز به دست آمد. کاهش راندمان حذف COD برای غلظت‌های فوق نیز مشاهده شد؛ به طوری که راندمان حذف COD در غلظت‌های فوق به ترتیب 9/68، 5/70، 4/54 و 8/33 درصد به دست آمد. اثرات بازدارندگی اتیلن‌دی‌کلراید بر میزان متان‌سازی بیومس بی‌هوازی از غلظت‌های بالاتر از 750 میلی‌گرم بر لیتر آغاز گردید. میزان متان تجمعی در غلظت‌های 750، 5000 و 20000 میلی‌گرم بر لیتر اتیلن‌دی‌کلراید به ترتیب 7/30، 86/9 و 9/1 میلی‌لیتر بود. نتایج آنالیز راندمان حذف اتیلن‌دی‌کلراید با استفاده از دستگاه GC در پایان دوره در غلظت‌های 500، 2000 و 3000 میلی‌گرم بر لیتر به ترتیب برابر 2/95، 7/94 و 8/93 درصد به دست آمد. نتیجه‌گیری: افزایش غلظت اتیلن‌دی‌کلراید از حدی به بالا باعث کاهش درصد تولید متان و بازدارندگی بر فعالیت باکتری‌ها می‌شود؛ به طوری که غلظت‌های بالاتر از 750 میلی‌گرم بر لیتر اثر بازدارندگی بر فعالیت متان‌سازی داشت.

Title: ارزیابی ریسک فاکتورهای ارگونومیکی وظایف تکراری در شاغلان صنایع دستی شهر اصفهان با استفاده از روش ارزیابی وظایف تکراری (ART)

http://hsr.mui.ac.ir/index.php/jhsr/article/view/2390

مقدمه: متأسفانه صنایع کوچک با وجود انجام مطالعات بسیار در رابطه با اختلالات اسکلتی- عضلانی شغلی و صنعتی ایران، کمتر مورد بررسی و ارزیابی قرار گرفتند. مطالعه حاضر با هدف ارزیابی ریسک فاکتورهای ارگونومیکی شاغلان صنایع دستی شهر اصفهان به وسیله یکی از جدیدترین روش‌ها، یعنی روش ارزیابی وظایف تکراری (Assessment of Repetitive Tasks یا ART) در سال 1393 اجرا گردید. روش‌ها: 100 نفر از شاغلان صنایع دستی وارد این تحقیق توصیفی- تحلیلی و مقطعی به روش انتخاب در دسترس شدند. ابزارهای مورد استفاده شامل پرسش‌نامه‌های دموگرافیک (محقق ساخته)، نقشه بدن (Body map یا BM) و روش ART بود. داده‌ها با به کارگیری آزمون‌های آماری t، One way ANOVA و ضریب همبستگی Pearson در نرم‌افزار SPSS آنالیز گردید. یافته‌ها: کارگران در گستره سنی 80-18 سال قرار داشتند. سابقه‌ کار افراد نیز به طور متوسط 38/14 ± 57/12 سال گزارش شد. داده‌های حاصل از BM نشان داد که 74 نفر (74 درصد) افراد، حداقل در 1 عضو بدن خود احساس درد ناشی از کار داشتند. تعداد اندام‌های دارای درد با سن و سابقه کار دارای ارتباط معنی‌دار بود (05/0 P <). میانگین و انحراف استاندارد نمره نهایی ART دست راست و چپ به ترتیب 30/11 ± 45/31 و 24/7 ± 32/24 به دست آمد. همچنین، 73 درصد از وظایف تکراری در سطح خطر بالا قرار گرفت. نتیجه‌گیری: صنایع دستی به خصوص با افزایش سن کارگران، دارای ریسک بالایی از نظر ایجاد اختلالات ترومای تجمعی در اندام‌های فوقانی بود. بنابراین، مداخله جهت ایجاد ایستگاه‌های کاری بهینه، طراحی مناسب ابزارها، توجه به ساعات کاری، رژیم استراحت و در نهایت ایجاد آگاهی لازم برای نحوه صحیح انجام کار توصیه می‌گردد.

Title: بررسی پراکنش گاز فرمالدهید در شهر زرقان به روش غیر فعال، با استفاده از نرم‌افزار سامانه اطلاعات جغرافیایی (GIS)

http://hsr.mui.ac.ir/index.php/jhsr/article/view/1837

مقدمه: هدف از انجام پژوهش حاضر، تعیین الگوی پراکنش گاز فرمالدهید در شهر زرقان به روش غیر فعال، با استفاده از نرم‌افزار GIS (Geographic information system) بود. روش‌ها: در اين مطالعه مقطعی، گاز فرمالدهيد در 10 ايستگاه مختلف توسط نمونه‌برداری غیر فعال اندازه‌گیری شد. نمونه‌برداری در یک فصل و به مدت 14 روز انجام شد. سپس، به کمک نرم‌افزار ArcGIS، در نقاط ديگر شهر به روش‌های مختلف نزدیک‌ترین همسایگی (Nearest neighbor یا NN)، Kriging و IDW (Inverse distance weighting) درون‌يابی صورت گرفت. یافته‌ها: در اطراف صنايع شيميايی فارس و سينا غلظت فرمالدهيدµgm-3 1/38 بود. درون‌يابی‌ها با GIS نشان داد که بسته به جهت وزش باد تا فاصله 1400 متری از منبع، غلظت اين آلاينده در حد مجاز µgm-3 10 می‌باشد. نتیجه‌گیری: تعیین غلظت فرمالدهید با درون‌يابی به روش نزديک‌ترين همسايگی مناسب‌تر بود. در هنگام پدیده وارونگی دمای صبحگاهی (Morning inversion)، در شرایط پایدار هوا در اطراف کارخانه، غلظت فرمالدهید چندين برابر حد میانگین بود. با وجود ارتفاعات اطراف شهر، غلظت فرمالدهید در مناطق مسکونی شهر زرقان و دودج در زمان نمونه‌برداری در حد استاندارد بود.

Title: مواجهه با ذرات معلق کمتر از 10 میکرون و تأثیر آن در بروز بیماری‌های تنفسی و قلبی- عروقی در هوای شهر اصفهان

http://hsr.mui.ac.ir/index.php/jhsr/article/view/2452

مقدمه: بحران آلودگی هوا در برخی شهرهای ایران از جمله اصفهان به دلیل شرایط خاص جغرافیایی، قرارگیری در یک گودال و تجمع مواد آلاینده در هوای سطحی، این شهر را به یکی از آلوده‌ترین شهرهای کشور از نظر میزان آلاینده‌های هوا تبدیل نموده است. هدف تحقیق حاضر، برآورد بیماری‌های قلبی- عروقی و تنفسی منتسب به 10PM (Particles matter up to 10 microns) در شهر اصفهان بود. روش‌ها: این مطالعه توصیفی در سال 1392 انجام شد. غلظت آلاینده 10PM با استفاده از دستگاه GRIMM پرتابل 15 کاناله (مدل 108، ساخت کشور آلمان)مورد اندازه‌گیری قرار گرفت. همچنین، پارامترهای دما و فشار از طریق دستگاه‌های سازمان هواشناسی ساعت به ساعت مشاهده و ثبت گردید. سپس اين داده‌ها در نرم‌افزار Excel پردازش شد و ميزان اثرات بهداشتي به وسیله تجزیه و تحلیل آماری و با استفاده از مدل Air Q سازمان بهداشت جهاني به دست آمد. یافته‌ها: غلظت 10PM در فصل گرم سال نسبت به فصل سرد سال (پاییز و زمستان) بیشتر بود. حداکثر غلظت 10PM، 83/1000 میکروگرم بر متر مکعب در فصل تابستان برای ایستگاه احمدآباد ثبت گردید. همچنین، تعداد تجمعي موارد بيماري‌هاي قلبی- عروقی و تنفسی شهر اصفهان در برآورد حد وسط خطر نسبي به ترتیب 585 نفر و 1517 نفر محاسبه شد. نتیجه‌گیری: بالا بودن تعداد موارد بیماری‌های تنفسی و قلبی- عروقی در شهر اصفهان به علت مواجهه با 10PM می‌تواند به دلیل میانگین بالاتر این ذرات و تداوم روزهای با غلظت بالای آن باشد.

Title: بررسی تأثیر ورزش‌های مقاومتی بر حوزه‌های عملکرد کاری و نمره افسردگی در افراد دارای تحصیلات دانشگاهی شهر اصفهان

http://hsr.mui.ac.ir/index.php/jhsr/article/view/2032

مقدمه: افسردگی نوعی اختلال خلق است که در آن فرد احساس غم، عدم لذت و دلسردی می‌کند. با توجه به افزایش بروز این اختلال در جامعه کنونی و به ویژه در افراد دارای تحصیلات دانشگاهی، هدف از انجام تحقیق حاضر، بررسی تأثیر ورزش‌های مقاومتی بر حوزه‌های عملکرد کاری و نمره افسردگی در افراد دارای تحصیلات دانشگاهی شهر اصفهان بود. روش‌ها: روش نمونه‌گيري در این پژوهش به صورت غیر احتمالی و در دسترس انجام شد. پرسش‌نامه‌های افسردگی و عملکرد کاری برای گروه‌های آزمودنی (40 نفر) و شاهد (40 نفر) قبل از انجام مداخله تکمیل گردید و تمرینات مقاومتی به مدت 8 هفته، هفته‌ای 3 روز و روزی 5/1 ساعت بر طبق پروتکل طراحی شده انجام گرفت. در نهایت، پرسش‌نامه‌ها در هفته اول پس از سپری شدن طول مدت مداخله اخذ گردید. یافته‌ها: بر اساس آزمون Paired t، تفاوت معنی‌داری در میزان عملکرد، رضایت از عملکرد و شدت افسردگی قبل و بعد از انجام مداخله در گروه آزمودنی مشاهده شد. همچنین، نتایج آزمون Independent t حاکی از وجود تفاوت معنی‌دار بین دو گروه در عملکرد، میزان رضایت از عملکرد و شدت افسردگی، بعد از انجام مداخله بود. جهت تعیین معنی‌داری داده‌ها، 001/ ≤ P در نظر گرفته شد. تجزيه و تحليل داده‌ها در نرم‌افزار SPSS انجام گردید. نتیجه‌گیری: انجام 8 هفته تمرینات مقاومتی می‌تواند به طور مؤثری منجر به کاهش شدت افسردگی و بهبود در عملکرد و میزان رضایت از عملکرد در بزرگسالان دارای تحصیلات دانشگاهی شهر اصفهان گردد.

Title: بررسي عوامل مؤثر بر مصرف خودسرانه دارو و الگوی مصرف داروهای مرتبط در بين دانشجويان دانشگاه علوم پزشکي تبریز در سال 1391

http://hsr.mui.ac.ir/index.php/jhsr/article/view/2005

مقدمه: هدف از انجام مطالعه حاضر، شناسایی عوامل مؤثر بر مصرف خودسرانه دارو در بین دانشجويان دانشگاه علوم پزشكي تبریز و عملكرد آن‌ها در اين زمينه بود. روش‌ها: این مطالعه توصيفي– مقطعي با مشارکت 385 نفر از دانشجویان دانشگاه علوم پزشکی تبریز اجرا گردید. داده‌ها از طریق پرسش‌نامه محقق ساخته جمع‌آوری شد. روایی پرسش‌نامه توسط 10 نفر از صاحب‌نظران بررسی و بعد از اعمال نظرات آنان، مورد تأیید قرار گرفت. پایایی پرسش‌نامه نیز با استفاده از همسانی درونی بررسی و تأیید گردید (83/0 α =). ارتباط بین متغیرهای کیفی با استفاده از آزمون‌های Fisher exact و 2c در نرم‌افزار SPSS بررسی شد. 05/0 > P به عنوان سطح معنی‌داری آزمون‌ها در نظر گرفته شد. یافته‌ها: میزان خوددرمانی در کل مطالعه 1/48 درصد (154 نفر) بود. همچنین، زنان بیشتر از مردان خوددرمانی انجام داده بودند (024/0 = P). ارتباط معنی‌داری بین رشته تحصیلی و میزان خوددرمانی مشاهده شد (035/0 = P). بیشترین علت خوددرمانی، تب و سردرد با 4/70 درصد، تجربه قبلی از بیماری با 6/41 درصد و خريد دارو از داروخانه در هنگام مصرف با 6/55 درصد بود. بیشترین داروهای مورد استفاده در خوددرمانی نیز به آنتی‌بیوتیک‌ها (60 درصد) و داروهای مربوط به سرماخوردگی (42 درصد) اختصاص داشت. نتیجه‌گیری: نتایج مطالعه نشان می‌دهد که میزان خوددرمانی در بین دانشجویان شیوع بالایی دارد. بنابراین، لازم است تلاش گردد تا با آموزش مناسب و اطلاع‌رسانی صحیح، مصرف خودسرانه داروها کاهش داده شود.

Title: بررسی تأثیر بسته آموزش الکترونیک بر آگاهي شیردهی مادران در دوره پس از زایمان

http://hsr.mui.ac.ir/index.php/jhsr/article/view/2034

مقدمه: فواید شناخته شده تغذیه با شیر مادر باعث شده است که سیاست‌های بهداشتی بر پایه ترویج تغذیه با شیر مادر بنا نهاده شود و به نظر می‌رسد که یکی از راه‌های مناسب ترویج شیردهی، ارتقای آگاهی از طریق آموزش الكترونيك باشد. مطالعه حاضر با هدف تعیین تأثیر بسته آموزش الکترونیک بر آگاهی نسبت به شیردهی در مادران انجام شد. روش‌ها: این مطالعه به شیوه نیمه تجربی سه گروهی و یک سو کور بر روی 300 زن نخست‌زا در دوره پس از زایمان در بیمارستان‌های زنان شهر ساوه در سال 1390 به روش نمونه‌گیری ساده صورت گرفت. یک گروه بسته آموزش الکترونیک به همراه آموزش چهره به چهره (گروه اول)، گروه دوم بسته آموزش الکترونیک به تنهایی (گروه دوم) و گروه شاهد تنها مراقبت‌های معمول (گروه سوم) را دریافت کردند. ابزار جمع‌آوری داده‌ها، پرسش‌نامه اطلاعات دموگرافیک و پرسش‌نامه شیردهی محقق ساخته (20 سؤالی) بود که در دو مرحله بلافاصله و سه ماه بعد از زایمان تکمیل گردید. تجزیه و تحلیل داده‌ها با استفاده از نرم‌افزار SPSS انجام شد. یافته‌ها: بر اساس نتایج آزمون آنالیز One way ANOVA، نمره آگاهی قبل از مداخله در هر سه گروه آموزشی تفاوت آماری معنی‌داری نداشت (127/0 = P)؛ در حالی که 3 ماه بعد از مداخله، تفاوت آماری معنی‌داری مشاهده شد (001/0 > P)، اما نمره آگاهی در گروه آموزش الکترونیک همراه آموزش چهره به چهره از نظر آماری با سایر گروه‌ها تفاوت معنی‌داري را نشان داد (001/0 > P). نتیجه‌گیری: آموزش الكترونيك همراه با آموزش چهره به چهره با افزایش آگاهی نسبت به شیردهی در ماه سوم پس از زایمان همراه بوده است.

Title: عوامل مؤثر بر بستري مجدد بيماران مبتلا به ديابت نوع 2 در بيمارستان‌هاي منتخب شهر کرمانشاه

http://hsr.mui.ac.ir/index.php/jhsr/article/view/2000

مقدمه: ديابت ملیتوس یکی از شایع‌ترین اختلالات متابولیک می‌باشد كه ششمین علت میرایی ناشی از بیماری‌ها را به خود اختصاص می‌دهد. در اين بين، بستري مجدد بيماران مبتلا به ديابت اهميت زيادي دارد و مي‌تواند هزينه زيادي را به سيستم سلامت تحميل نماید. از این‌رو، مطالعه حاضر به بررسي عوامل مؤثر بر بستري مجدد بيماران مبتلا به ديابت نوع 2 در بيمارستان‌هاي منتخب شهر کرمانشاه پرداخت. روش‌ها: این مطالعه از نوع توصیفی– تحلیلی بود که به صورت مقطعی در سال 1391 انجام شد. 140 بیمار مبتلا به دیابت با روش نمونه‌گیری در دسترس و از 3 بیمارستان امام خمینی (ره)، طالقانی و امام رضا (ع) انتخاب گردیدند. برای تعيين روايي پرسش‌نامه، روش اعتبار محتوي مورد استفاده قرار گرفت. پایایی پرسش‌نامه با ضریب Cronbach's alpha، 86 درصد به دست آمد. داده‌ها با استفاده از آزمون آماری 2c در نرم‌افزار SPSS تحلیل شد. یافته‌ها: آزمون آماری 2c رابطه معنی‌داری را بین میزان بستری مجدد با متغيرهاي جنسیت، سطح تحصیلات، نوع شغل، تعداد فرزندان، مصرف سیگار در حال حاضر، سابقه مصرف سیگار، سوء مصرف مواد و سابقه ابتلا به بیماری‌ها به دست آورد. نتیجه‌گیری: نتايج مطالعه حاضر تصوير روشني را از دلایل بستري مجدد بيماران مبتلا به ديابت نشان داد. مطالعات گذشته كه در كشور انجام شد، پديده بستري مجدد را بيشتر به صورت عام مورد بررسي قرار دادند. اين مطالعه با ارايه يك رويكرد تخصصي، امكان برنامه‌ريزي هرچه بهتر سياست‌گذاران را براي اين دسته از بيماران فراهم نمود تا بتوانند از بخش زيادی از هزينه‌هاي بستري مجدد جلوگيري كنند.

Title: نمونه‌گیری پاسخگو محور، رویکردی نوین در نمونه‌گیری از بیماری‌های نادر و پنهان

http://hsr.mui.ac.ir/index.php/jhsr/article/view/2485

مقدمه: مطالعه دقیق جمعیت‌های پنهان و با دسترسی سخت به منظور شناسایی خصوصیات آن‌ها بسیار با اهمیت است؛ چرا که سلامت جامعه را به دلیل داشتن رفتارهای پرخطر به مخاطره می‌اندازد. با این حال، نمونه‌گیری از این جوامع به دلایل عمده‌ای مانند پنهان بودن آن‌ها و نداشتن چارچوب دقیق از طریق روش‌های نمونه‌گیری متداول امکان‌پذیر نیست. روش‌ها: پژوهش حاضر  به تعریف مفاهیم کاربردی و نکات کلیدی در اجرای روش نمونه‌گیری پاسخگو محور پرداخته است.  در این نوع روشها تمرکز بر روی طرح‌هایی  است که هدف آن‌ها بررسی رفتارهای پرخطر و پنهان نظیر رفتارهای پرخطر جنسی، اعتیاد تزریقی و ابتلا به عفونت HIV  می‌باشد. یافته‌ها: از مزایای روش نمونه‌گیری پاسخگو محور، علاوه بر تولید برآوردهای نااریب و کارا برای پارامترهای مورد نیاز در جوامع در معرض خطر ابتلا به بیماری‌های نادر و پنهان، در مقایسه با سایر روش‌های نمونه‌گیری ارجاع زنجیره‌ای و متداول، نو‌آوری‌ در حین اجرای آن نیز می‌باشد. نتیجه‌گیری: استفاده از روش نمونه‌گیری پاسخگو محور به منظور برآورد دقیق و نااریب نرخ شیوع بیماری‌های نادر و پنهان، در برنامه‌ریزی‌های بهداشت و سلامت جامعه، پیشنهاد می‌شود.

Title: تأثیر روش‌های مختلف خشک کردن بر خصوصیات تغذیه‌ای قارچ خوراکی- دارویی Agaricus blazei

http://hsr.mui.ac.ir/index.php/jhsr/article/view/2626

مقدمه: قارچ Agaricus blazei، از مهم‌ترین قارچ‌های خوراکی- دارویی و حاوی املاح معدنی، ویتامین‌ها و آمینواسید‌های ضروری بدن می‌باشد. این قارچ به صورت‌های مختلف تازه‌خوری، محصول خشک شده و فرآورده‌های مختلف دارویی مورد استفاده قرار می‌گیرد. از مهم‌ترین فرایند‌های پس از برداشت در جهت افزایش کیفیت و مدت زمان نگهداری محصولات، خشک کردن می‌باشد. هدف از انجام این پژوهش، یافتن بهترین روش خشک کردن به منظور حفظ بهتر خواص تغذیه‌ای و دارویی این قارچ بود. روش‌ها: در این پژوهش، تأثیر دو نوع خاک پوششی کمپوست برگشتی و ورمی‌کمپوست به همراه روش‌های خشک کردن از طریق آون، مایکروویو و انجماد خشک، بر ظرفیت آنتی‌اکسیدانی، میزان بازجذب آب و میزان فیبر رژیمی قارچ دارویی بلازئی مورد ارزیابی قرار گرفت. پژوهش حاضر به صورت فاکتوریل در قالب طرح كامل تصادفي اجرا گردید. یافته‌ها: بر طبق نتایج، بیشترین ظرفیت آنتی‌اکسیدانی و فیبر رژیمی در روش مایکروویو مشاهده گردید؛ در حالی که کمترین ظرفیت آنتی‌اکسیدانی نمونه‌ها در روش آون، در بستر حاوی کمپوست برگشتی قارچ مشاهده شد. بیشترین میزان بازجذب آب نیز در روش انجماد خشک در هر دو تیمار خاک پوششی و کمترین میزان در روش آون در بستر حاوی ورمی‌کمپوست به دست آمد. نتیجه‌گیری: روش‌های مختلف خشک کردن، تأثیرات متفاوتی را بر کیفیت قارچ بلازئی نشان داد که با توجه به هدف مورد نظر (خصوصیات تغذیه‌ای، ظاهری و صرفه اقتصادی)، می‌توان از هر یک از روش‌های فوق در جهت افزایش کیفیت قارچ بلازئی استفاده نمود.

Title: تعیین خطر نسبی بروز سرطان مری در شهرستان‌های ایران با استفاده از مدل چند سطحی بیزی مکانی- زمانی

http://hsr.mui.ac.ir/index.php/jhsr/article/view/2328

مقدمه: میزان بروز استاندارد شده‌ سنی سرطان مری در ایران برای زنان در رتبه پنجم و برای مردان در رتبه هشتم قرار دارد. در مطالعه حاضر، با توجه به  ضرورت آگاهی از چگونگی توزیع جغرافیایی این سرطان، نقشه‌بندی بروز این سرطان در سطوح استانی و شهرستانی ترسیم شده است. روش‌ها: پژوهش  حاضر یک مطالعه کاربردی اکولوژیک  است . جامعه مورد مطالعه از تمام بیماران مبتلا به سرطان مری بین سال‌های 1384 تا 1386 در ایران تشکیل شده است. جهت برآورد خطر نسبی سرطان مری، از مدل چند سطحی بیزی مکانی- زمانی‌ استفاده گردیده است. تجزیه‌ و تحلیل داده‌ها با استفاده از رویکرد بیزی INLA (Integrated nested Laplace approximation) در نرم‌افزار R انجام شد. یافته‌ها: تعداد کل سرطان مری ثبت شده طی سال‌های 86-1384 در ایران 7160 مورد بوده است. بر اساس نتایج مدل سه سطحی با تعدیل تمامی عوامل خطر، روند تغییرات سالانه معنی‌دار و  و به صورت افزایشی بوده است. نتیجه‌گیری: با توجه به برازش مدل سه سطحی با تعدیل عوامل خطر فعالیت فیزیکی و مصرف سیگار، مصرف میوه‌ها و سبزیجات و اضافه وزن، توصیه می‌شود که در مطالعات آینده از مدل‌های چند سطحی با تعدیل عوامل خطرساز مؤثر بیشتری استفاده شود. این مطالعه نشان داد که مناطق شمال شرقی، شمال غربي و بعضی از نقاط مرکزی ایران دارای بیشترین خطر بروز سرطان مری می‌باشد.

Title: استراتژی مدیریت سبز برای سیستم‌های انرژی، فاضلاب و کنترل آلاینده‌های هوا در بیمارستان‌های آموزشی شهر یزد در سال 1392

http://hsr.mui.ac.ir/index.php/jhsr/article/view/2028

مقدمه: با کاربرد فرایندهای مدیریت استراتژیک، می‌توان در سطح محلی به طراحی و توسعه سیستم‌‌های بهینه انرژی‌، فاضلاب و حفاظت از محیط‌ زیست در مقابل آلاینده‌های هوا اقدام نمود. این فرایند تنها با توجه به نقاط قوت، ضعف، فرصت‌ها و تهدیدهای بیمارستان‌ها ممکن می‌شود. پژوهش حاضر با هدف ارایه استراتژی‌های بهینه مدیریت سبز در رابطه با سیستم‌های یاد شده با استفاده از مدل SWOT (Strength, Weakness, Opportunity, Threaten) در بیمارستان‌های آموزشی شهر یزد انجام شد. روش‌ها: این پژوهش به لحاظ هدف، از نوع کاربردی و روش تحقیق آن توصیفی- تحلیلی بود. روند پژوهش با تکمیل چک‌لیست، مصاحبه با کارشناسان و مدیران دانشگاه و بیمارستان‌های آموزشی از مهر تا اسفند سال 1392 صورت گرفت. برای تحلیل یافته‌ها از ماتریس‌های ارزیابی عوامل داخلی و خارجی و برای تدوین استراتژی‌ها از مدل راهبردی SWOT استفاده گردید. یافته‌ها: استراتژی‌های پیشنهاد شده جهت مدیریت سبز سیستم‌ها به ترتیب SO، WO، ST و WT می‌باشند. در این رابطه طرح‌های مدیریت استراتژیک سبز SO، بر ارایه طرح قابل‌ اجرایی در جهت کاستن از مصرف حامل‌های انرژی و آب، فراهم‌سازی تکنولوژی‌های نو به‌ منظور بهینه‌سازی شیوه مصرف آب و انرژی، توجه به رعایت تمامی نکات در زمینه استفاده مجدد از فاضلاب یا لجن حاصل از تصفیه فاضلاب، توسعه تمام مفاد قانون نحوه جلوگیری از آلودگی هوا و تدوین گزارش سالانه از اقدامات لازم برای تحقق مدیریت سبز در بخش‌های بیمارستان‌های آموزشی تأکید نمود. نتیجه‌گیری: تمرکز بر استراتژی SO در راستای حداکثر استفاده از نقاط قوت‌ داخلی و فرصت‌های خارجی موجود، باید در اولویت مدیران بیمارستان‌های آموزشی و استان جهت مدیریت سبز و ارتقای کیفیت خدمات بهداشتی و درمانی قرار گیرد.

Title: بررسی رابطه بین خودکارامدی با مصرف وعده‌های غذایی در دختران دانش‌آموز دارای اضافه وزن و مبتلا به چاقی مدارس راهنمایی شهر شاهین‌شهر

http://hsr.mui.ac.ir/index.php/jhsr/article/view/2223

مقدمه: شیوع اضافه وزن و ابتلا به چاقی در کودکان و نوجوانان سراسر جهان به میزان قابل توجه و هشدار دهنده‌ای رسیده است. عدم مصرف منظم وعده‌های غذایی یکی از مهم‌ترین علل چاقی در کودکان و نوجوانان می‌باشد. مطالعات گذشته، یکی از مهم‌ترین فاکتورهای روان‌شناختی مؤثر در تغییر رفتارهای تغذیه‌ای نوجوانان را خودکارامدی معرفی کرد. از این‌رو، هدف از انجام مطالعه حاضر، بررسی رابطه بین خودکارامدی با مصرف وعده‌های غذایی دانش‌آموزان دختر دارای اضافه وزن و مبتلا به چاقی شهر شاهین‌شهر بود. روش‌ها: این مطالعه مقطعی بر روی 172 نفر دانش‌آموز دختر دارای اضافه وزن و مبتلا به چاقی شهر شاهین‌شهر در سال تحصیلی 93-1392 صورت گرفت. پس از نمونه‌گیری تصادفی ساده و اندازه‌گیری شاخص‌های تن‌سنجی، پرسش‌نامه مربوط به خودکارامدی و مصرف وعده‌های غذایی توسط دانش‌آموزان تکمیل گردید. یافته‌ها: میانگین سنی و شاخص توده بدنی (Body mass index یا BMI) دانش‌آموزان شرکت کننده به ترتیب 6/0 ± 4/13 و 6/3 ± 2/28 به دست آمد. BMI با مصرف صبحانه و میان وعده‌های صبح و عصر دارای ارتباط معکوس و معنی‌داری بود (001/0 > P). ارتباط مثبت و معنی‌داری بین خودکارامدی با مصرف صبحانه و میان وعده‌های صبح و عصر مشاهده شد (001/0 > P). نتیجه‌گیری: خودکارامدی به طور مؤثری مصرف صبحانه دانش‌آموزان مبتلا به چاقی را پیش‌بینی کرد. به نظر می‌رسد که جهت درک بهتر رابطه بین سازه‌های روان‌شناختی با مصرف صبحانه، مطالعات بیشتری در آینده به خصوص مطالعات مداخله‌ای نیاز است.

Title: ارزیابی نحوه مديريت زباله‌هاي دندان‌پزشكي شهر ياسوج، ایران

http://hsr.mui.ac.ir/index.php/jhsr/article/view/2268

مقدمه: مديريت بهينه زباله‌هاي دندان‌پزشكي به دليل نقش عمده آن‌ها در انتشار عوامل بيماری‌زا و تهديد سلامت انسان و محيط ‌زيست، از اهميت ويژه‌ای برخوردار است. روش‌ها: جامعه مورد بررسی مطالعه توصیفی- مقطعی حاضر، 40 مرکز دندان‌پزشکی بود که 11 مرکز از طریق نمونه‌گیری ‌تصادفی انتخاب شد. نمونه‌برداری در 3 نوبت طی 3 روز کاری متوالی (دوشنبه، سه‌شنبه و چهارشنبه) از هر مرکز صورت گرفت. این مواد به 5 گروه پسمانده‌های پاتولوژیک، شیمیایی- دارویی، عفونی، شبه‌خانگی و نوک‌تیز و برنده تقسیم‌ گردید و با ترازوی‌ آزمایشگاهی ‌توزین شد. یک چک‌لیست حاوی 25 سؤال جهت ارزیابی نحوه مدیریت پسماندهای‌ دندان‌پزشکی مورد استفاده قرار گرفت. یافته‌ها: بیشترین میانگین وزنی برای پسماند عفونی (1920 گرم) و کمترین آن برای بخش پاتولوژیک (32 گرم) به دست آمد. بیشترین میانگین وزنی ‌سرانه پسماند مربوط‌ به زباله‌های عفونی با 43 گرم و بعد از آن زباله‌های شیمیایی- دارویی با 23/15 گرم به ازای هر نفر محاسبه شد. زباله‌های عفونی با 60 درصد و در رتبه بعد زباله‌های شیمیایی- دارویی با 21 درصد، بیشترین درصد سرانه فصلی تولید زباله را به خود اختصاص دادند. نتیجه‌گیری: نتایج مطالعه حاضر نشان داد که برنامه‌ای جهت کاهش تولید زباله در بیشتر مراکز مورد مطالعه وجود نداشت. در رابطه با مدیریت بهینه زباله‌های تولیدی، پیشنهاد می‌گردد که علاوه بر آموزش دندان‌پزشکان جهت انجام فعالیت‌های کاهش، جداسازی‌ و ‌بازیافت ‌در داخل مطب، جمع‌آوری، حمل و ‌نقل و دفع هر کدام از اجزای زباله به صورت جداگانه و مطابق با دستورالعمل‌های مربوط انجام گیرد.
